# Supplementary material for: Inflammatory Cellular Response to Mechanical Ventilation in Elastase-Induced Experimental Emphysema: Role of Preexisting Alveolar Macrophages Infiltration
Source: Biomed Res Int. 2018 Dec 19;2018:5721293. doi: 10.1155/2018/5721293 (PMC6313972; doi:10.1155/2018/5721293)

SUPPLEMENTARY MATERIALS

INFLAMMATORY CELLULAR RESPONSE TO MECHANICAL VENTILATION IN ELASTASE-INDUCED EXPERIMENTAL EMPHYSEMA: ROLE OF PRE-EXISTING ALVEOLAR MACROPHAGES INFILTRATION

Anahita ROUZE, Guillaume VOIRIOT, Elise GUIVARCH, Françoise ROUX, Jeanne TRAN VAN NHIEU, Daniel ISABEY, Laurent BROCHARD, Bernard MAITRE, Armand MEKONTSO-DESSAP, and Jorge BOCZKOWSKI

METHODS

Animal model

All the experiments were performed in accordance with the official regulations of the French Ministry of Agriculture and the US National Institute of Health guidelines for the experimental use of animals, were approved by the local institutional animal care and use committee, and were conducted in a specific "little animal" plateform (Plateforme Exploration Fonctionnelle du Petit Animal, INSERM U955, Créteil, France). Six-weeks-old male C57BL/6 mice (Janvier Labs, Le Genest Saint-Isle, France) were used. Under anesthesia, mice received the instillation of either 5 IU of porcine pancreatic elastase (Elastin Products, Owensville, MO, USA) diluted in 50 µl of saline (Elastase mice), or 50 µl of saline (Saline mice) into the surgically exposed trachea. Mice were then subjected to subsequent ventilation at two time points, 14 and 21 days after instillation.

Mechanical ventilation

Mice were anesthetized with intraperitoneal thiopental (30 µg/g of body weight; Hospira, Meudon-La-Forêt, France) followed by continuous inhaled 5% isoflurane (Abbott, Rungis, France). The larynx was surgically exposed and the trachea was intubated orally under direct vision with a metal cannula (internal diameter of 1 mm, Harvard Apparatus, Les Ulis, France). Mice in mechanical ventilation (MV) group were ventilated in the supine position using humidified gas (20mgH_2O/L absolute humidity, MR410 humidifier, Fischer & Paykel Healthcare, Courtaboeuf, France), with a Vt of 8 µL/g of body weight, a respiratory rate of 180 /min, an inspiratory to expiratory time ratio of 67%, an end-expiratory pressure of 1.5 cmH_2O, and a fraction of inspired oxygen of 0.4 - 0.6, by means of a computer-driven small-animal ventilator (flexiVent, Scireq, Montreal, Canada), as previously reported. The tracheal cannula was properly secured with surgical thread (Ethicon 3-0, Ethicon, Auneau, France) before connection to mechanical ventilator in order to avoid leaks. The cervicotomy was closed with surgical thread (Ethicon 6.0, Ethicon, Auneau, France). Mechanical ventilation lasted two hours with continuous anesthesia maintained by 1.5% inhaled isoflurane and muscle paralysis using intraperitoneal pancuronium at the onset of the experiment (0.8 µg/g of body weight; Organon, Puteaux, France) to ensure passive mechanical conditions. The body temperature was monitored using a rectal probe and was maintained at 36.5°C with a blanket connected to a homeothermic regulator (Homeo-blanket system 50-7221F, Harvard Apparatus, Les Ulis, France). An intraperitoneal warm fluid bolus of 20 µL/g of saline was performed immediately after the induction of anesthesia. A control group (SV) consisted of anesthetized, intubated mice, maintained on spontaneous ventilation for two hours, under conditions identical to MV mice, except for muscle paralysis. At the end of the timed ventilator protocol, mice were exsanguinated via sectioning the left carotid artery. Arterial blood was obtained, using a heparinized needle and syringe, then transported in ice rapidly after collection, for determination of blood gases, using a GEM Premier 3000 analyser (Instrumentation Laboratory, Lexington, MA, USA) in D21 mice. Blood gases data were similar between the 4 groups at D21 (n=10-13/group), allowing us to validate fluid replacement strategy, and both ventilator

settings in MV mice, and anesthesia depth in SV mice (table S1). To note, blood gases analysis were not performed in D14 mice, and the validity of the experimental protocol has been extrapolated to this group.

TABLE S1. Arterial blood gases in SV and MV mice, 21 days after saline or elastase tracheal instillation.

	**Saline SV**	**Elastase SV**	**Saline MV**	**Elastase MV**
**PaO_2/FiO_2**	450 ± 100	371 ± 124	417 ± 99	460 ± 84
**$PaCO_2$**, mmHg	27 ± 7	34 ± 21	34 ± 10	30 ± 6
**pH**	7.31 ± 0.08	7.30 ± 0.16	7.23 ± 0.09	7.28 ± 0.12
**HCO_3^-**, mmol/L	14.6 ± 3.7	15.1 ± 5.1	16.4 ± 3.9	15.3 ± 7.0
**Lactates**, mmol/L	3.2 ± 2.4	3.2 ± 3.0	2.8 ± 0.7	2.5 ± 1.0

Definition of abbreviations: PaO2, partial pressure of oxygen in arterial blood; PaCO2, partial pressure of carbone dioxide in arterial blood; SV, spontaneous ventilation; MV, mechanical ventilation.
Values are expressed as median ± interquartile range; n = 10-13 animals /group.
No significant difference was found between groups. Ventilator settings (MV mice) and anesthesia depth (SV mice) allowed suitable minute ventilation.

Respiratory mechanics

Special features of the *flexiVent* ventilator include a continuous monitoring of airway pressures and a precision computer-controlled piston that is capable of accurately measuring the delivered volume (with appropriate corrections for gas compression) and to produce any desired waveform, allowing respiratory mechanics assessment with the forced oscillation technique and pressure-volume curves. Before each mouse was connected to the ventilator, pressure and piston displacement calibration data were collected to correct the respiratory mechanics data. Mice were allowed to stabilize on the ventilator for 5 minutes and were then inflated three times to a transrespiratory pressure of 30 cmH2O to establish a standard volume history. Forced oscillation technique were assessed at initiation of mechanical ventilation, before (H0) and after (H0’) volume history standardization (recruitment maneuver), and then repeated hourly (H1 and H2), to capture the time course and the detailed response to mechanical ventilation. Respiratory system dynamic resistance, elastance, and compliance were calculated in the *flexiVent* software by fitting the single frequency forced oscillation technique data (a 1.2 second, 2.5 Hz single-frequency signal called SnapShot-150 perturbation) to the single compartment model. In addition, a continuous pressure-volume curve (pressure-driven from 3 to 30

cmH2O) was performed at start and end of mechanical ventilation. The loops were processed by the *flexiVent* software to fit the Salazar-Knowles equation and to calculate parametric results, such as the quasi-static compliance of the respiratory system.

Specimen collection

After sacrifice by exsanguination via sectioning the left carotid artery, two sets of samples collection were performed. In a first set of experiments, a thoracotomy was immediately performed, exposing heart and lungs. Pulmonary circulation was flushed with 2 mL of saline directly injected into the right ventricle after ablation of the left atrium. Lungs were then harvested, fixed in 4% paraformaldehide by tracheal injection at a constant transpleural pressure of 20cmH_2O, held in 4% paraformaldehide and finally embedded in paraffin. In a second set of experiments, bronchoalveolar lavage (BAL) was first performed with two separate 1 mL aliquots of saline at 20°C injected through tracheal tube. Then immediately following thoracotomy, pulmonary circulation was identically flushed (to remove residual blood within pulmonary vasculature and reduce nonadherent leukocytes from subsequent flow cytometric analysis), and both lungs were collected for flow cytometry analysis.

Morphometric analysis

Great-axis sagittal sections of 5 µm thickness of the left lung were cut in a systematic fashion and were stained with hematoxylin and eosin. Five digital photomicrographs were acquired from the cranial, medial, and caudal regions of each slide at x200 magnification, excluding areas where large bronchi or vessels predominated, resulting in a total of 15 images per lung (Axioplan 2 microscope *equipped with an MRc digital color camera (Zeiss, Oberkochen, Germany)).* Emphysema was quantified by measurement of alveolar diameters with an image analysis software (ImageJ, NIH, Bethesda, USA). This automated analysis was made vertically and horizontally on each photomicrograph. The mean chord length of alveoli was obtained by averaging those measurements.

Bronchoalveolar lavage

The total cell count of BAL was determined for a fresh fluid specimen using a Malassez hemocytometer (Hycor biomedical, Indianapolis, IN, USA). BAL fluid was centrifuged (1500 rpm, 15 min at 4°C). The cell pellet was diluted in saline, and differential cell counts were done on cytocentrifuge preparations (Cytospin3; Shandon Scientific, Cheshire, UK) stained with Diff-Quick stain (Baxter Diagnostics, McGaw Park, IL, USA).

Flow cytometric analysis

Preparation of lung cell suspensions

Freshly collected lungs were immediately placed in a storage medium consisting of RPMI 16/40 supplemented with 0.1% L-glutamine and 0.1% nonessential amino acids and kept on ice. Mechanical disruption followed by enzymatic digestion of murine lungs were then performed, as previously described. Lung tissue, placed in previously described medium, supplemented with 5% fetal bovine serum (FBS), 0.35 mg/mL collagenase XI (Sigma-Aldrich, Saint Louis, MO, USA) and 0.1 mg/mL type IV bovine pancreatic DNAse (Sigma-Aldrich), was cut into small pieces by rapid chopping using a scalpel. The dissected tissue was then incubated at 37°C with 5% CO2 during 35 minutes. Enzymatic action was stopped by adding 9 mL of previous medium storage containing 10% FBS, and digested lungs were further disrupted by gently pushing the tissue through a 70 µm nylon cell strainer. Cell suspensions were then centrifuged at 900 rpm. To lyse contaminating red blood cells, the cell pellet was incubated during 5 minutes at room temperature with 5 ml of ACK Lysing Buffer (NH4Cl and KHCO3, ThermoFisher Scientific Waltham, MA, USA). Cells were then washed with saline, recentrifuged, and the pellet resuspended in 4 ml of saline. Cell counts were performed using a Malassez hemocytometer after Trypan blue staining. Cell suspensions were lastly centrifuged and resuspended in saline in order to obtain approximately 10 millions cells per mL. In cytometry

dedicated tubes, 500,000 cells (50 µL) were incubated with 50 µL of anti-mouse CD16/CD32 (Fc Block, 10µg/mL, clone 2.4G2, BD Biosciences, San Jose, CA, USA) at 4°C during 15 minutes, to prevent nonspecific binding of antibodies to the Fc receptors, and then stained with fluorochrome-conjugated anti-mouse antibodies for CD11b, CD11c, Gr1 (Ly6C/G), F4/80 and CD62L (L-selectin) or appropriate isotype-matched controls. All antibodies used are listed in table S2. Finally, cytometry tubes were centrifuged, cell pellets resuspended in 500 µL saline with 1% formaldehyde, and tubes were stored away from light at 4°C.

TABLE S2. Fluorochrome conjugated anti-mouse antibodies.

Surface molecule	Fluorochrome	Clone	Isotype control	Manufacturer
**Ly6G/Ly6C (Gr1)**	FITC	RB6-8C5	Rat IgG2a, κ	BD Biosciences
**Ly6G/Ly6C (Gr1)**	PE/Cy7	RB6-8C5	Rat IgG2b, κ	Biolegend
**CD11c**	PE	HL3	Hamster IgG1, λ2	BD Biosciences
**F4/80**	PE/Cy7	BM8	Rat IgG2a, κ	Biolegend
**CD62L**	APC	MEL-14	Rat IgG2a, κ	Biolegend
**CD11b**	APC/Cy7	M1/70	Rat IgG2b, κ	BD Biosciences

BD Biosciences, San Jose, CA, USA; Biolegend, San Diego, CA, USA

Identification of inflammatory cell populations

Cells were analysed the following day by a 7channel cytometer (CyAn ADP Analyser, Beckman Coulter, Brea, USA). One channel was dedicated to measurement of cell autofluorescence. Complete data analysis was performed with Summit software (Summit v4.3, Dako, Cambridge, UK). Three inflammatory cell populations in murine lung were identified (figure S1). CD11b-, CD11c+ phenotype, with high autofluorescence defined alveolar macrophages (figure S1c). Monocytes and neutrophils were identified as CD11b+, CD11c- cells but differ in their granularity (side-scatter, SS), and F4/80 and Gr1 expression. High SS and F4/80-, Gr1+ phenotype defined neutrophils, whereas low SS, and F4/80+, Gr1*mid* phenotype characterized monocytes (figures S1d, S1e, S1f). Cells activation state were assessed using expression of CD62L and CD11b adhesion molecule, as well as Gr1. All flow cytometric results were presented as relative values, called percentage of gated cells. Indeed, our cytometer did not allow automated simultaneous counting of collected lung cells, resulting in inaccurate absolute values.

Figure S1. Flow cytometric analysis of different inflammatory cell populations in mouse lungs. **a.** Dot-plot showing side-scatter (SS) versus forward-scatter (FS) within total cell population. Cellular debris and lymphocyte were excluded from the analysis. Myeloid cells were gated (G1) on compatible size (FS) and granularity (SS). **b.** Dot-plot showing FS versus pulse width within G1 gated events. Doublets and triplets were subtracted from cell analysis through a second gate (G2) defined on pulse width, which distinguished cell aggregates from unique events (called singlets). From there, only combined G1 and G2 gated events were analysed. **c.** Dot-plot showing CD11c versus CD11b expression within combined G1 and G2 gated events. Alveolar macrophages were identified by CD11c+ CD11b- phenotype. Autofluorescence level was high in this cell population. **d.** Dot-plot SS versus CD11b expression within combined G1 and G2 gated events. G3 gated CD11b+ cells with neutrophils compatible granularity, G4 gated CD11b+ cells with monocytes compatible granularity. **e.** Dot plot CD11c versus Gr1 expression within combined G1, G2 and G3 gated events. Neutrophils were defined as CD11c- Gr1+ cells. **f.** Dot plot CD11c versus Gr1 expression within combined G1, G2 and G4 gated events. Monocytes were defined as CD11c- Gr1*mid* cells.

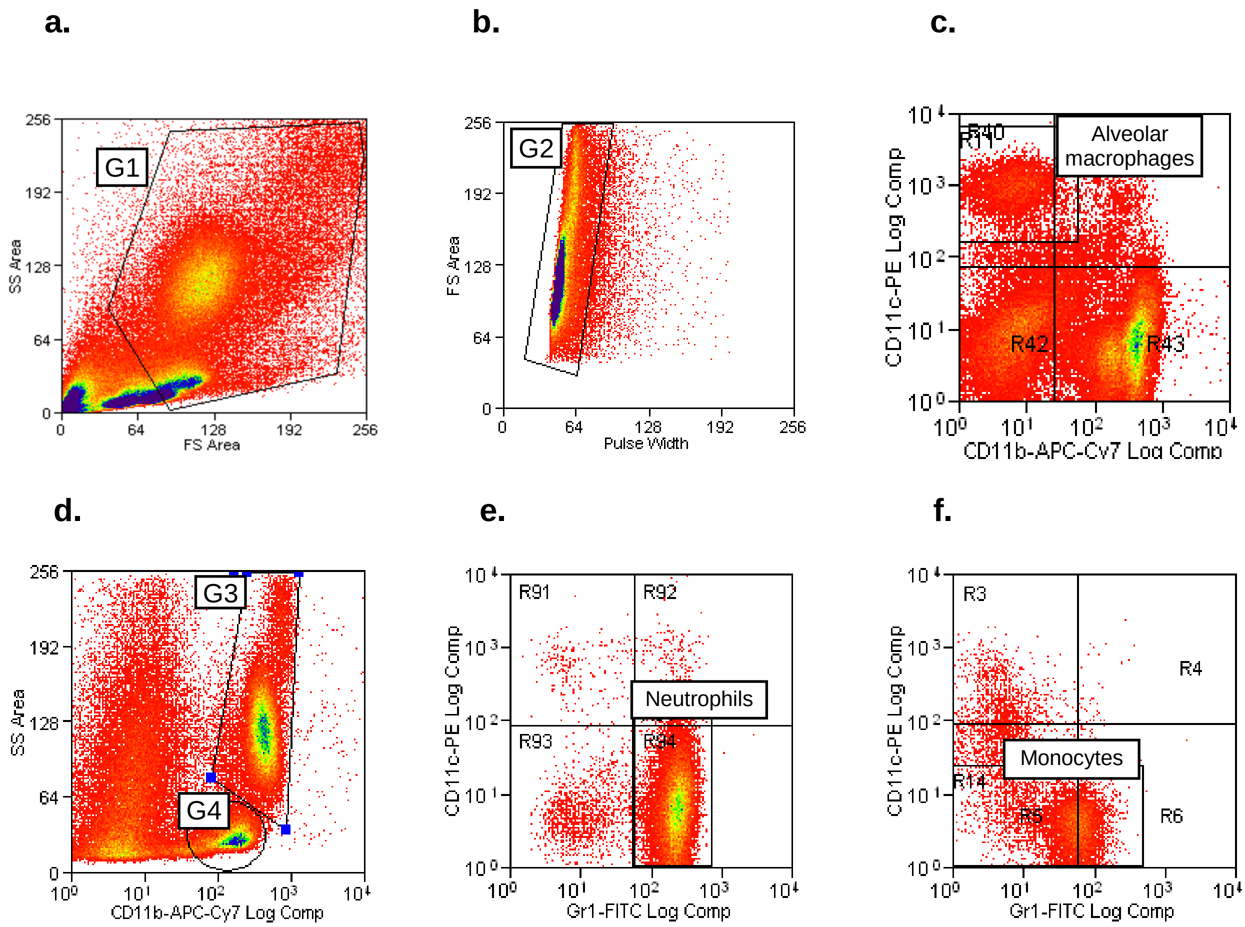

Supplement: Supplementary Materials — Additional information about the methods are available in supplementary material file, including arterial blood gases data, fluorochrome-conjugated anti-mouse antibodies used, and flow cytometric analysis of inflammatory cell populations in mouse lungs. [file 5721293.f1.pdf]
